# BMP-2/6 Heterodimer Is More Effective than BMP-2 or BMP-6 Homodimers as Inductor of Differentiation of Human Embryonic Stem Cells

**DOI:** 10.1371/journal.pone.0011167

**Published:** 2010-06-17

**Authors:** Elvira Valera, Michael J. Isaacs, Yasuhiko Kawakami, Juan Carlos Izpisúa Belmonte, Senyon Choe

**Affiliations:** 1 Structural Biology Laboratory, Salk Institute, La Jolla, California, United States of America; 2 Joint Center for Biosciences, Incheon, Korea; 3 Gene Expression Laboratory, Salk Institute, La Jolla, California, United States of America; Katholieke Universiteit Leuven, Belgium

## Abstract

**Background:**

Bone Morphogenetic Protein (BMP) signaling pathways are involved in differentiation of stem cells into diverse cell types, and thus BMPs can be used as main guidance molecules for in vitro differentiation of human stem cells.

**Methodology/Principal Findings:**

We have analyzed the ability for inducing differentiation of the heterodimer BMP-2/BMP-6 (BMP-2/6) compared to the homodimers BMP-2 or BMP-6, using human embryonic stem (hES) cells H9 as model system. When incubated in a medium with high concentration of basic fibroblastic growth factor (FGF2), 100 ng/ml of human recombinant BMPs induced morphological changes and differentiation of hES cells in 24 to 48 hours. After 5 days, expression of differentiation markers was induced and quantified by quantitative PCR (qPCR) and flow cytometry. BMP-2/6 exhibited stronger activity for the induction of the expression of trophectodermal (*CDX2*) and endodermal (*SOX17, GATA4, AFP*) markers than BMP-2 or BMP-6 homodimers. BMP-2/6 also induced the expression of *BMPR2* gene more effectively than BMP-2 or BMP-6 when used at the same concentration and time. Moreover, the percentage of cells expressing the surface endodermal marker CXCR4 was also increased for the heterodimer when compared to both homodimers. BMP-2/6 was a more potent activator of Smad-dependent (SMAD1/5) and Smad-independent signaling (mitogen-activated protein kinases ERK and p38) than BMP-2 and BMP-6, and the activation of these pathways might play a role in its increased potency for inducing hES cell differentiation.

**Conclusions/Significance:**

Therefore, we conclude that BMP-2/6 is more potent than BMP-2 or BMP-6 for inducing differentiation of hES cells, and it can be used as a more powerful substitute of these BMPs in *in vitro* differentiation guidance.

## Introduction

Embryonic stem (ES) cell lines derived from the epiblast tissue of the inner cell mass of a blastocyst or earlier morula stage embryos are pluripotent and can develop to the three primary germ layers: ectoderm, endoderm and mesoderm. For that reason, human embryonic stem (hES) cells represent an excellent clinical source of precursor cells for cell-based therapy if they can be guided to differentiate into a certain cell type needed to treat damaged tissue in chronic diseases or injuries [1]. In addition to tissue regeneration, hES cell derivatives can be used in studies of cellular development, tumorigenesis, and in the discovery or cytotoxicity screening of new drug candidates [2].

Different members of the Transforming Growth Factor β (TGFβ) family have been implicated in various developmental stages and processes. One subfamily, the Bone Morphogenetic Proteins (BMPs), have been traditionally studied with regard to bone and cartilage development, but recently their effects have been studied in mouse and human stem cell cultures [3]. Murine and human stem cells display differences in their behavior upon incubation with BMPs. BMP signaling promotes self-renewal in mouse stem cells when incubated together with Leukemia Inhibitory Factor in the absence of serum replacement or conditioned medium [4]. In contrast, inhibition of BMP signaling is a requirement for long term maintenance of hES cells [5], while incubation with BMPs is a potent inductor of differentiation of these cells in conditions that would otherwise support self-renewal [6].

The diverse members of the BMP subfamily exert different effects on hES cells. BMP-2 and BMP-6 have been involved in osteogenesis of human mesenchymal stem cells [7], [8] and BMP-2 is also known to induce extraembryonic endoderm differentiation of hES cells [9]. BMP-4 has been linked to mesoderm and endoderm formation, as well as kidney and bone development. In culture, BMP-4 induces trophoblast differentiation of hES cells [10], and endoderm differentiation of monkey stem cells [11].

From a structural point of view, the mature segment of the BMPs is highly conserved in all organisms and contains seven cysteine amino acid residues. Six of these residues are involved in the formation of intrachain disulphide bonds that forms a rigid “cysteine-knot” molecular structure, and the seventh cysteine residue is involved in the formation of a dimer via interchain disulphide bond. BMPs interact with a pair of type II and type I receptors (e.g. BMP receptor type Ia, BMPR1A; Activin receptor-like kinase 1, ACVRL1; BMP receptor type 2, BMPR2; Activin receptor type IIa, ACVR2A; Activin receptor type IIb, ACVR2B), which are also respectively structurally conserved [12]–[14]. Most of TGFβ family ligands, including BMPs, are produced as either homo- or heterodimers, increasing the variability of the effector molecule. Although most studies in the past have been performed with BMP homodimers, mainly due to their availability, there are natural heterodimers with equal, if not increased, bioactivity [15]–[19], and the heterodimer activity is not well elucidated yet.

We have recently established a procedure that allows us to generate large quantity of BMP-2/BMP-6 heterodimer (BMP-2/6) by a chemical refolding method [20]. This methodology enabled us to study the activity of the BMP-2/6 heterodimer in a variety of experiments that require sub-milligram amount of ligands. The heterodimer showed a higher affinity to both receptor types than its homodimeric counterparts, and increased SMAD1-dependent signaling activity by luciferase reporter assay, osteogenic differentiation-inducing activity in mouse MC3T3-E1 cells and chondrogenic activity in primary cultured embryonic limb mesenchyme [20]. To study its biological activity on hES cells, we have compared BMP-2, BMP-6 and BMP-2/6 in inducing differentiation of hES cells, quantified by gene expression analysis of specific differentiation markers. We found that among BMP-2, BMP-6 and BMP-2/6, BMP-2/6 is more effective than BMP-2 or BMP-6 for inducing both trophoblast and endoderm differentiation of hES cells H9. BMP-2/6 also induced higher levels of SMAD1/5 phosphorylation, and increased activation of Smad-independent signaling pathways (ERK and p38 mitogen-activated protein/MAP kinases) that might be related to its increased potency for inducing hES cell differentiation. Our results support the hypothesis of an enhanced biological activity of the BMP-2/6 heterodimer, and these characteristics make BMP-2/6 a good candidate for its application in *in vitro* differentiation guidance of human stem cells.

## Results

### BMP-2, BMP-6 and BMP-2/6 induce differentiation of hES cells in a dose- and type-dependent manner

H9 cells were cultured in mTeSR1 (StemCell Technologies) on Growth Factor-Reduced (GFR) Matrigel-coated wells (BD Biosciences). Forty-eight hours (h) after splitting, hES cells were treated with BMP-2, BMP-6 or BMP-2/6 at 100 ng/ml in mTeSR1 for 5 days, and the time course of morphological changes was analyzed. Morphological changes were observed as soon as 24 h after beginning incubation, and evident differentiation morphology appeared usually after another 24 h.

**Figure 1:**
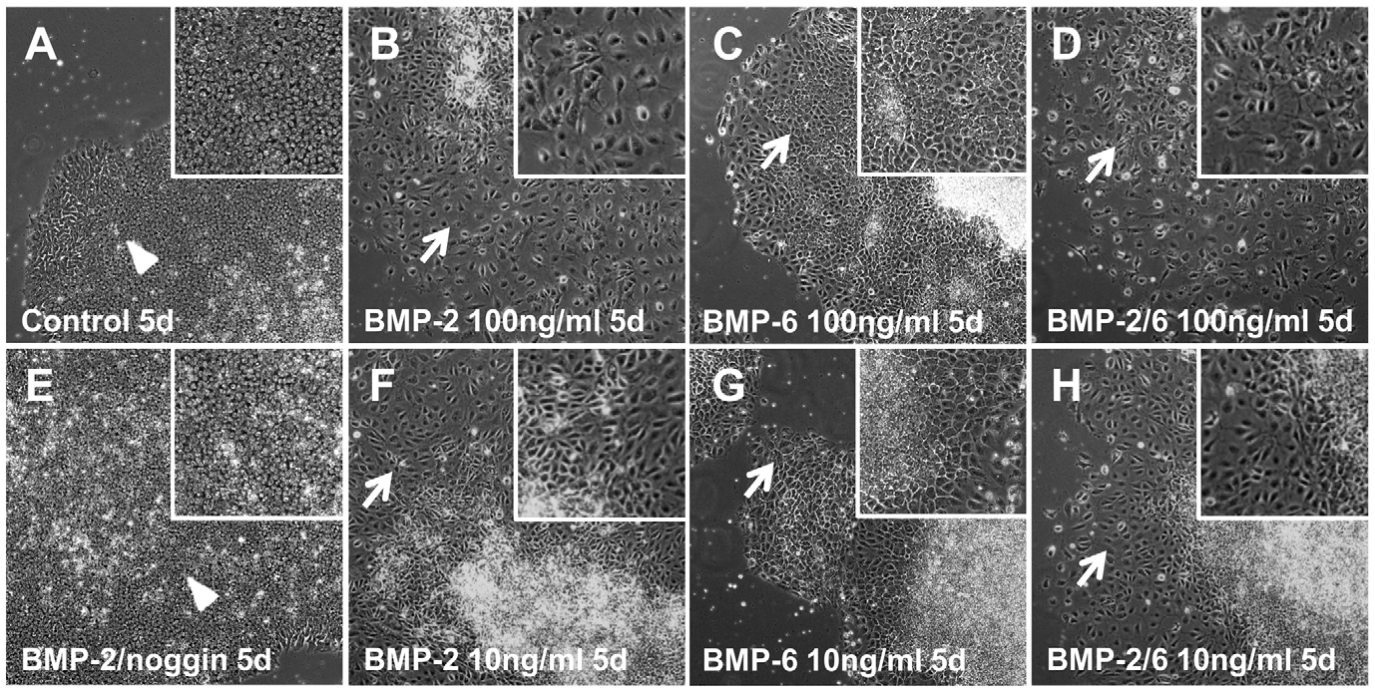
Morphological changes of hES cells after treatment with BMPs. H9 cells were treated with BMP-2, BMP-6 or BMP-2/6 in mTeSR1 for 5 days. Noggin 1 µg/ml was used as antagonist to BMP-2. Medium was changed daily for fresh medium supplemented with the desired concentration of agonists. Pictures were taken using an inverted microscope and 10X objective. A, control cells in mTeSR1. B, BMP-2 100 ng/ml. C, BMP-6 100 ng/ml. D, BMP-2/6 100 ng/ml. E, BMP-2 100 ng/ml + noggin 1 µg/ml. F, BMP-2 10 ng/ml. G, BMP-6 10 ng/ml. H, BMP-2/6 10 ng/ml. Arrowheads indicate morphologically pluripotent cells; arrows point out morphologically differentiated areas. Insets belong to 3× magnification.

Morphological changes induced by BMPs started synchronically at the periphery of the colonies and spread towards the center. Usually a central core of highly packed, morphologically undifferentiated cells remained after 5 days of treatment (Figure 1). In comparing this morphology to spontaneous differentiation morphology, BMP-treated cells were usually bigger and homogeneously shaped, while spontaneous differentiation tended to be restricted to discrete spots, not synchronic and morphologically heterogeneous (data not shown).

The time course of morphological changes depended on the BMP ligand used as inductor of differentiation. BMP-2 and BMP-2/6 at 100 ng/ml induced morphological changes after 24 h and evident differentiation morphology of hES H9 cells after 48 h, whereas the same concentration of BMP-6 only induced similar morphological changes after an additional 24 h in comparison to BMP-2 or BMP-2/6. There was no increased activity of BMP-2/6 when compared to BMP-2 in the number of differentiated colonies, rate of differentiation or morphology. As expected, noggin at 1 µg/ml completely blocked the morphological changes induced by BMP-2 at 100 ng/ml (Figure 1E), which confirms that the differentiation is BMP-dependent.

Lower concentrations of agonists (1 and 10 ng/ml) were also tested. BMPs at 1 ng/ml did not induce any morphological change on hES cell colonies after 5 days of incubation (data not shown). BMP-2 or BMP-2/6 at 10 ng/ml induced morphological changes, but with a delay of 24 h when compared to 100 ng/ml (Figure 1F, 1H). BMP-6 at 10 ng/ml only elicited modest morphological changes after 5 days, indicating again the lower potency of BMP-6 to induce differentiation of H9 cells in our culture conditions (Figure 1G). Based on morphological examination, BMP-2/6 heterodimer and BMP-2 homodimer are comparably active, whereas BMP-6 homodimer is not as active.

### Gene expression analysis of BMP-treated hES cells: BMP-2 and BMP-2/6 show similar activity to induce loss of pluripotency markers

In order to examine the differentiation by BMP-2/6 with molecular markers, we performed quantitative gene expression analysis. After 5 days of incubation with BMPs at 100 ng/ml, total RNA was extracted following the guanidinium thiocyanate-phenol-chloroform protocol [21]. 5 µg of total RNA were used for the reverse transcription reaction and gene expression was analyzed by quantitative PCR (qPCR) with specific primers for pluripotency or differentiation markers. We selected *GAPDH* as housekeeping gene to perform the normalization of qPCR results as *GADPH* expression is known to be less affected in hES cell differentiation than other normalization standards [22]. All the results were also normalized to non-treated H9 cells grown in mTeSR1 (control conditions).

**Figure 2:**
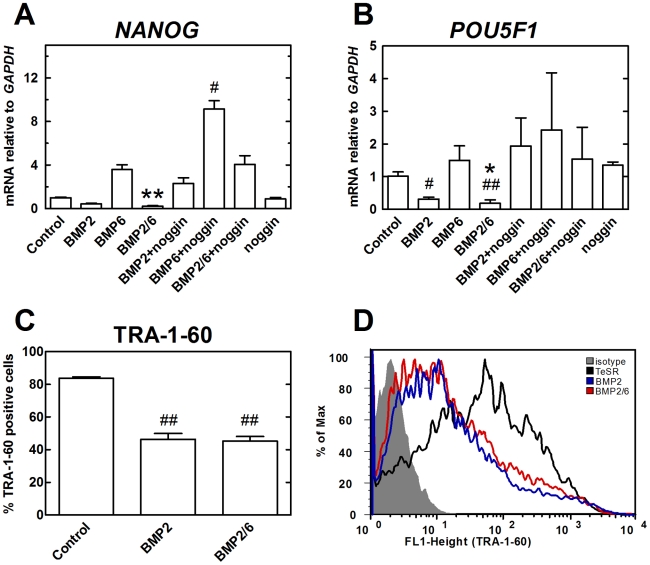
Expression analysis of stem cell markers after treatment with BMPs. H9 cells were treated with BMP-2, BMP-6 or BMP-2/6 at 100 ng/ml in mTeSR1 for 5 days. Noggin 1 µg/ml was used as antagonist. After 5 days of treatment, qPCR and flow cytometry were used to analyze expression of stem cell markers. qPCR values correspond to relative expression compared to *GAPDH* mRNA. As control, cells growing in mTeSR1 were used. Treatments were repeated at least in three different experiments, and results are expressed as average ± SD. A, qPCR analysis of *NANOG* expression. B, qPCR analysis of *POU5F1* expression. C, Flow cytometry analysis of the embryonic stem cell marker TRA-1-60. D, Flow cytometry histogram corresponding to the results shown in C. (*) P<0.05 BMP-2 vs. BMP-2/6; (**) P<0.01 BMP-2 vs. BMP-2/6; (#) P<0.05 control vs. BMP; (##) P<0.01 control vs. BMP.

Consistent with the morphological change, expression of stem cell markers *NANOG* and *POU5F1/OCT4* were reduced in BMP-2-treated H9 cells, and even more efficiently in BMP-2/6-treated H9 cells (Figure 2A, 2B). In contrast, expression of these markers was not reduced in BMP-6 treated cells, despite the obvious differentiation morphology in a significant percentage of cells after 5 days (Figure 1C). Interestingly, BMP-6 induced an increase in the expression of *NANOG*, which was potentiated by co-incubation with noggin but not induced by noggin alone (Figure 2A).

We also quantified the percentage of cells which lost the embryonic stem cell marker TRA-1-60 (Figure 2C, 2D). H9 cells were exposed to 100 ng/ml of BMP-2 or BMP-2/6 for 5 days, at which point TRA-1-60 was tagged with an Alexa Fluor 488 conjugated antibody. Cells were analyzed by flow cytometry to quantify the percentage of TRA-1-60-positive cells. We observed a 50% loss of TRA-1-60-positive pluripotent cells after BMP-2 or BMP-2/6 treatment (Figure 2C), suggesting that differences in the expression of stem cell markers do not necessarily reflect the percentage of pluripotent cells.

### Gene expression analysis of BMP-treated hES cells: BMP-2 and BMP-2/6 differ in their ability to induce the expression of differentiation markers

**Figure 3:**
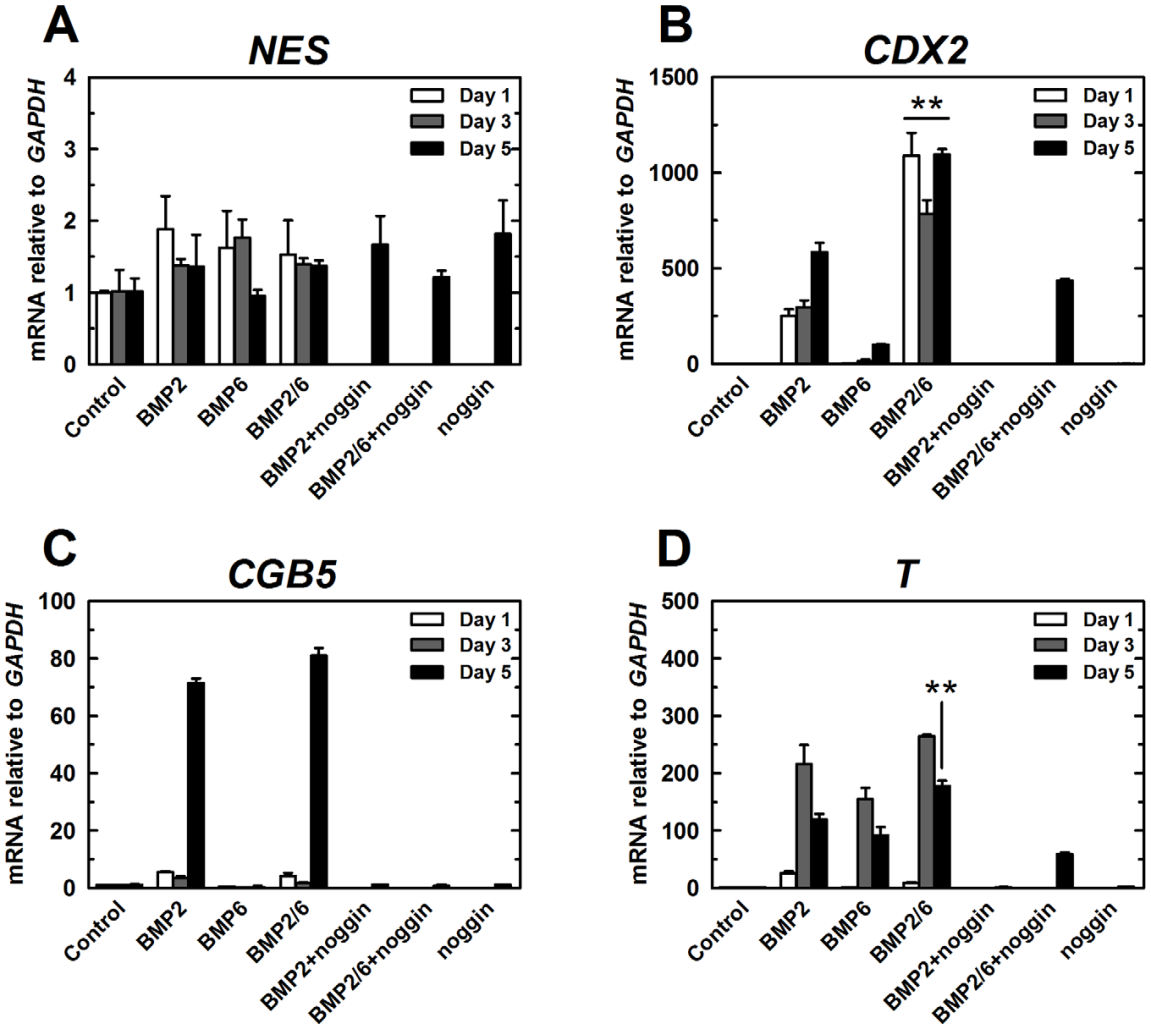
qPCR expression analysis of differentiation markers after treatment with BMPs (I). H9 cells were treated with BMP-2, BMP-6 or BMP-2/6 at 100 ng/ml in mTeSR1 for 1, 3 or 5 days. Noggin 1 µg/ml was used as antagonist. After 5 days of treatment, qPCR was used to analyze expression of differentiation markers. qPCR values correspond to relative expression compared to *GAPDH* mRNA. As control, cells growing in mTeSR1 were used. Treatments were repeated at least in three different experiments, and results are expressed as average ± SD. A, *NES*. B, *CDX2*. C, *CGB5*. D, *T*. (**) P<0.01 BMP-2 vs. BMP-2/6.

Next, we examined markers for differentiation after 1, 3 and 5 days of BMP treatment. There were no significant variations in the expression levels of *NES/NESTIN* (Figure 3A), which is a marker of neural tissue. This confirms previous reports that activation of BMP signaling blocks neuroectodermal differentiation of hES cells [9], [23], [24].

We examined next trophectodermal markers *CDX2* and *CGB5/HCG* [25], [26]. Expression of these markers was highly increased by BMP-2 treatment, and this change was completely blocked by noggin at 1 µg/ml (Figure 3B, 3C). BMP-6 also induced *CDX2* expression, but no *CGB5* expression. Interestingly, BMP-2/6 was better inducer of *CDX2* marker than BMP-2 or BMP-6 at any time point analyzed, revealing an increased activity of the heterodimeric form in inducing trophoblast differentiation.

Expression of the early stage mesendodermal marker *T* (human brachyury homolog) was induced by BMP-2, BMP-6 and BMP2/6 (Figure 3D). *T* was expressed in BMP-2 and BMP-6-treated cells at the same level. Similar to the induction of trophectodermal markers, BMP-2/6 showed stronger activity as inducer of this marker. *T* is a hallmark of both definitive mesoderm cells as cells undergoing meso-endoderm transition [27]. *T* expression peaked after 3 days of treatment and decreased afterwards, which suggests that its expression is mainly a prelude of endoderm differentiation. Similar to the pattern of *CDX2* expression, noggin at 1 µg/ml completely blocked *T* expression induced by BMP-2, but only partially blocked the increase induced by BMP-2/6 (Figure 3B, 3D).

### Gene expression analysis of BMP-treated hES cells: BMP-2/6 shows stronger activity to induce endodermal marker expression

Given the strong induction of *T*, an early mesendodermal marker, we further characterized the differentiation induced by BMPs by the analysis of endodermal markers. *SOX17* and *CXCR4* are markers of definitive endoderm systematically used in studies of endoderm differentiation [28]–[30]. Nevertheless, *SOX17* can also be expressed in extraembryonic endoderm, and *CXCR4* can also be expressed in mesendoderm cells, so a combination of several markers is the best choice when definitive endoderm needs to be accurately quantified. *GATA4*, *GATA6* and *AFP* are extraembryonic endodermal markers [31], and *AFP* is also a marker of hepatic differentiation of hES cells [32].

**Figure 4:**
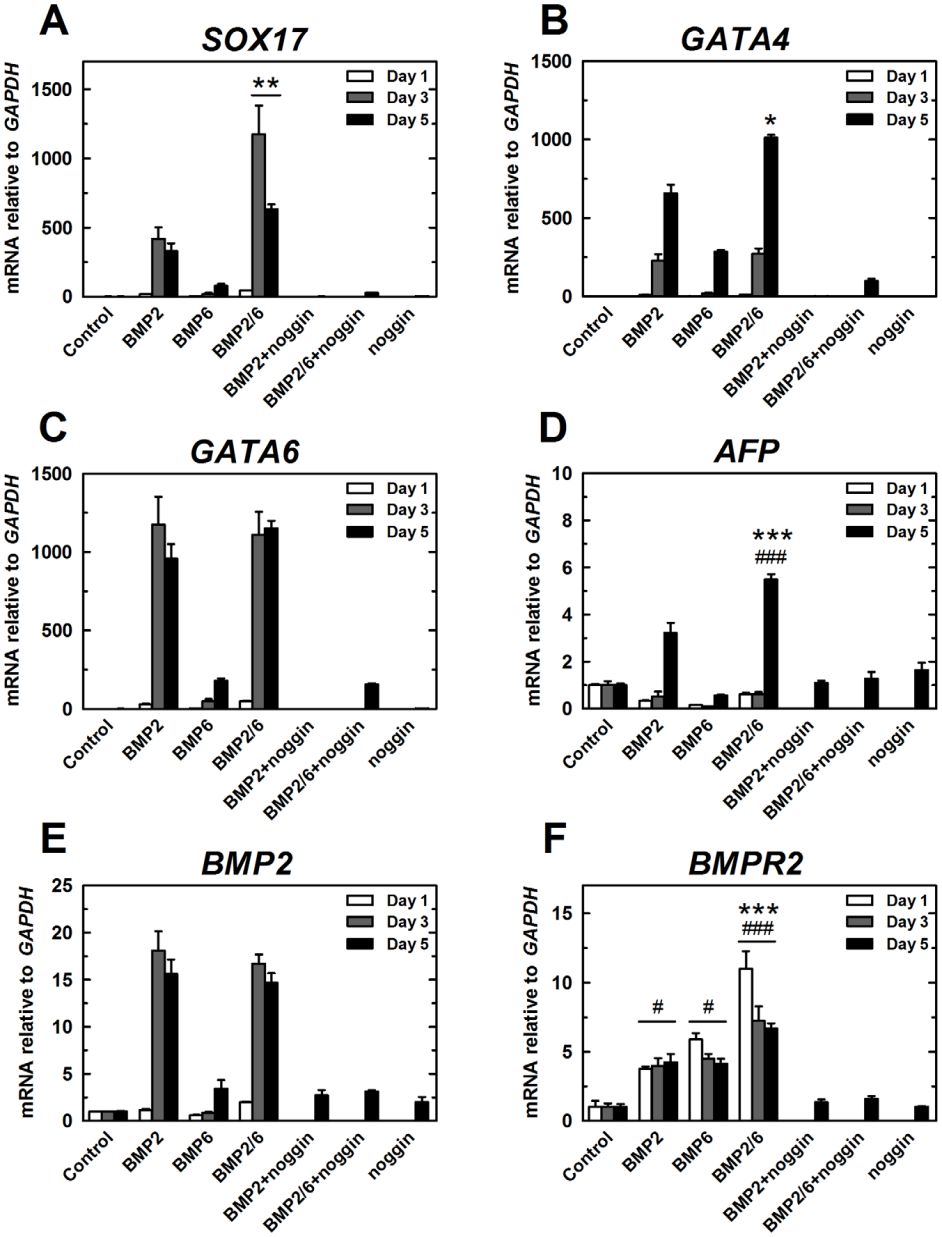
qPCR expression analysis of differentiation markers after treatment with BMPs (II). H9 cells were treated with BMP-2, BMP-6 or BMP-2/6 at 100 ng/ml in mTeSR1 for 1, 3 or 5 days. Noggin 1 µg/ml was used as antagonist. After 5 days of treatment, qPCR was used to analyze expression of differentiation markers. qPCR values correspond to relative expression compared to *GAPDH* mRNA. As control, cells growing in mTeSR1 were used. Treatments were repeated at least in three different experiments, and results are expressed as average ± SD. A, *SOX17*. B, *GATA4*. C, *GATA6*. D, *AFP*. E, *BMP2*. F, *BMPR2*. (*) P<0.05 BMP-2 vs. BMP-2/6; (**) P<0.01 BMP-2 vs. BMP-2/6; (***) P<0.001 BMP-2 vs. BMP-2/6; (#) P<0.05 control vs. BMP; (##) P<0.01 control vs. BMP; (###) P<0.001 control vs. BMP.

The pattern of expression of the endodermal markers *SOX17*, *GATA4*, *GATA6* and *AFP* indicated that BMP-2/6 is an effective inducer of these markers and BMP-6 is not (Figure 4A-D). Expression of *SOX17*, *GATA4* and *GATA6* showed a strong induction under our conditions (500 to 1000 times *GAPDH* expression), while expression of *AFP* showed a modest level of induction. From these results we conclude that BMP-2/6 is a more efficient inductor of endodermal differentiation than BMP-2 in our experimental paradigm.

We also analyzed the expression of several members of the BMP signaling pathway: agonists (*BMP2*, *BMP6*) and receptors (*BMPR1A/ALK3*, *BMPR1B/ALK6, BMPR2*, *ACVR1/ALK2, ACVR2A*, *ACVR2B*). Expression of *BMP2* gene was induced by BMP-2 and BMP-2/6 treatment, suggesting a positive feedback in the BMP signaling pathway (Figure 4E). *BMP2* is also a mesoderm marker, indicating that BMP-2 and BMP-2/6 could also induce modest levels of mesoderm differentiation. Expression of type II receptor *BMPR2* was also increased in all the conditions, but BMP-2/6 was again the most effective inducer of this gene (Figure 4F). The expression levels of *BMPR1A*, *BMPR1B, ACVR1, ACVR2A, ACVR2B* and *BMP6* were, however, not significantly affected by any of the treatments (Figure S1). Therefore, these results suggest that the positive feedback induced by BMP-2 or BMP-2/6 comprises an increase in the expression of both agonist (*BMP2*) and receptor (*BMPR2*).

With these results combined, we conclude that BMP-2, BMP-6 and BMP-2/6 induce mainly trophoblast and endoderm differentiation in our experimental conditions. These BMPs also regulate the expression of *BMP2* and *BMPR2* genes, suggesting the possibility of a positive feedback of stimulation of BMP-2 signaling pathway. The level of expression of selected genes (endodermal *SOX17*, *GATA6*, *GATA4*, *AFP* and BMP signaling members *BMP2* or *BMPR2*) can be used as a measure of the biological activity of different BMPs in H9 hES cells. It is interesting to note that BMP-2/6 is a more effective inducer of expression of differentiation markers than BMP-2 or BMP-6, even though it is a heterodimeric assembly of BMP-2 and BMP-6, and BMP-6 is not an active inducer of differentiation.

### Gene expression analysis of BMP-treated hES cells: BMP-2 and BMP-2/6 differ in their ability to induce definitive endoderm marker CXCR4

**Figure 5:**
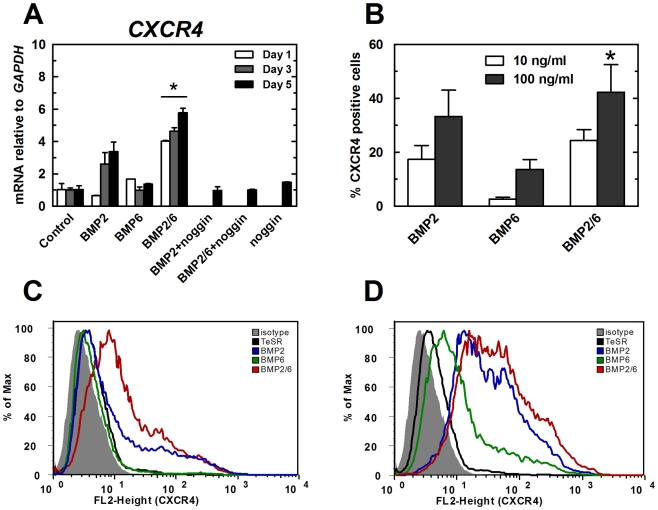
Expression analysis of endodermal marker CXCR4 in hES cells treated with BMPs. H9 cells were treated with BMP-2, BMP-6 or BMP-2/6 in mTeSR1 for 1, 3 or 5 days. Noggin 1 µg/ml was used as antagonist. After 5 days of treatment, qPCR and flow cytometry were used to analyze expression of the endodermal marker *CXCR4*. qPCR values correspond to relative expression compared to *GAPDH* mRNA. As control, cells growing in mTeSR1 were used. Treatments were repeated at least in three different experiments, and results are expressed as average ± SD. A, qPCR analysis of *CXCR4* expression. B, Flow cytometry analysis of CXCR4 after incubation with BMP-2, BMP-6 or BMP-2/6 at 10 or 100 ng/ml. C, D, Flow cytometry histograms of CXCR4-positive cells after incubation with BMPs at 10 (C) or 100 ng/ml (D). As negative controls, untreated cells (TeSR) and PE-conjugated mouse IgG2a isotype control (isotype) were used. (*) P<0.05 BMP-2 vs. BMP-2/6.

*CXCR4* is a marker of definitive endoderm cells and a surface receptor that has been used to purify this population of cells without compromising their viability [33]. We decided to measure *CXCR4* gene expression levels and the percentage of the CXCR4-positive population to quantify definitive endoderm induction after BMP treatments. H9 cells were treated with BMP-2, BMP-6 or BMP-2/6 at 100 ng/ml for 1, 3 and 5 days in mTeSR1, and RNA was extracted and analyzed by qPCR. While the increase in the expression of *CXCR4* was modest compared to the increase of extraembryonic endodermal markers, we observed stronger activity of BMP-2/6 in inducing this marker than that of the homodimers BMP-2 and BMP-6 (Figure 5A).

This increase in mRNA levels could not be directly correlated to changes in the percentage of definitive endodermal cells. To quantify the percentage of CXCR4-positive endoderm cells present after incubation with BMPs, we used flow cytometry in non-permeabilization conditions. H9 cells were exposed to 10 or 100 ng/ml of BMP-2, BMP-6 or BMP-2/6 for 5 days and CXCR4 was targeted with a Phycoerythrin (PE) labeled antibody. Cells were analyzed by flow cytometry and the percentage of CXCR4-positive cells quantified (Figure 5B–D). As expected, the percentage of CXCR4-positive cells depended on the BMP and its concentration (Figure 5B). When incubated at 10 ng/ml, BMP-2 induced an average of 17.4% CXCR4-positive cells and BMP-2/6 induced 24.4%. When incubated at 100 ng/ml, BMP-2/6 also induced the expression of CXCR4 in more cells that BMP-2 (42.3% and 33.3% respectively). Nevertheless, BMP-6 had reduced differentiation potency when compared to BMP-2 and BMP-2/6 (2.6% at 10 ng/ml and 13.6% at 100 ng/ml).

These results confirm that BMP-2/6 is better inducer of definitive endoderm differentiation of H9 cells than BMP-2 or BMP-6 when measured by the level of expression of endodermal markers and percentage of CXCR4-positive cells. BMP-2/6 induced the expression of CXCR4 marker in 1.3 times more cells than BMP-2, similar value to the ratio obtained by qPCR analysis (average of 1.6 times). Those results confirm that BMP-2/6 is a better inducer of endoderm differentiation than BMP-2 by a factor of 30–60% in this model system.

### Signaling pathways analysis of BMP treated hES cells: BMP-2/6 is a better inductor of Smad-dependent and Smad-independent signaling

Finally, we analyzed the involvement of different signaling pathways in the early stages of BMP-2, BMP-6 and BMP-2/6-induced differentiation of hES cells. Activation of BMP receptors leads to the recruitment and phosphorylation of the receptor regulated Smads (R-Smads) SMAD1, SMAD5 or SMAD8, that in complex with SMAD4 migrate into the nucleus and activate the transcription of specific target genes [34]–[36]. But BMPs and other TGFβ family members also activate a multitude of intracellular signaling pathways in addition to Smads to regulate cell function, including MAP kinases [34], [37], [38].

**Figure 6:**
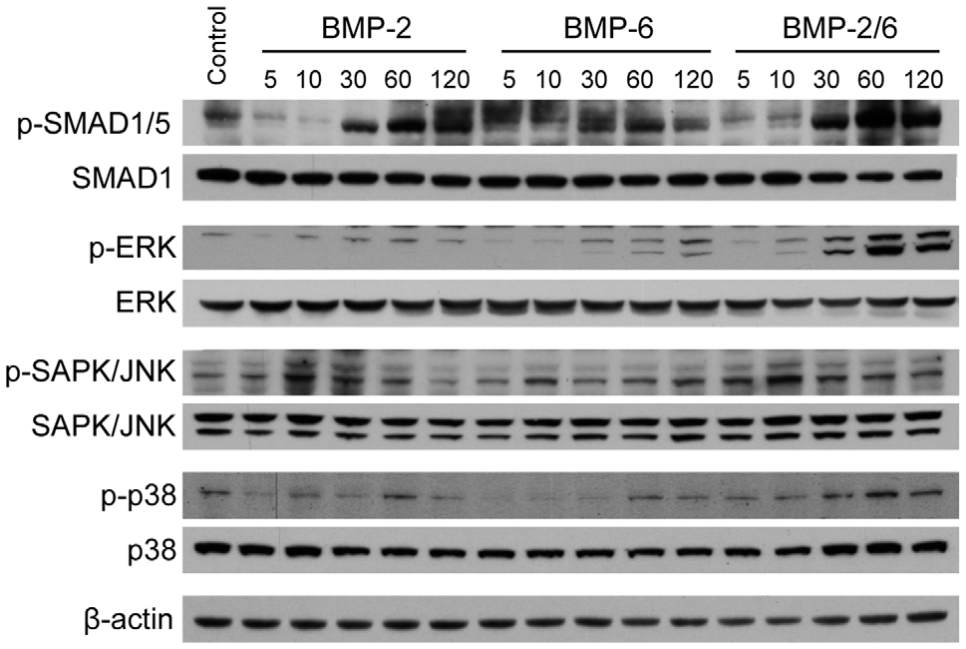
Activation of Smad-dependent and Smad-independent signaling pathways by BMPs in hES cells. H9 cells were treated with BMP-2, BMP-6 or BMP-2/6 at 100 ng/ml in mTeSR1 for 5, 10, 30, 60 and 120 min, or with mTeSR1 for 120 min (Control). Cell lysates were immunoblotted using different antibodies for phosphorylated (p-, active) and total forms of transcription factors or MAP kinases. β-actin was used as loading control.

H9 cells were treated with BMP-2, BMP-6 or BMP-2/6 at 100 ng/ml for 5, 10, 30, 60 or 120 min in mTeSR1, and protein was extracted and analyzed by immunoblotting (Figure 6). Antibodies against phosphorylated (active) and total forms of different members of Smad-dependent and Smad-independent signaling pathways were used. As expected, all three BMPs induced SMAD1/5 phosphorylation in a time dependent manner. BMP-2/6 induced higher levels of SMAD1/5 phosphorylation than BMP-2 or BMP-6 after 1–2 h of treatment, while BMP-6 was the less potent activator at any time point (Figure 6).

Next we analyzed activation of MAP kinase pathways by BMPs. Levels of phosphorylated ERK (pan ERK) increased after 30 min of treatment with BMPs (Figure 6), and BMP-2/6 strongly induced ERK activation when compared to the homodimers BMP-2 and BMP-6. This effect could also be observed in the case of p38 MAP kinase, a known member of BMP Smad-independent signaling cascades. After 2 h of treatment, both proteins remained phosphorylated at high levels (Figure 6). On the contrary, phosphorylation of SAPK/JNK was similar in BMP-2 and BMP-2/6 treated cells and started decreasing after 30–60 min of treatment (Figure 6).

Therefore the BMP-2/6 heterodimer induced higher levels of SMAD1/5 phosphorylation and it is also a more potent activator of Smad-independent signaling pathways than the homodimeric forms BMP-2 or BMP-6, and these properties might be related to its increased potency for inducing differentiation of hES cells.

### FGF2 and TGFβ1 components of the culture medium affect how hES cells respond to BMP treatment

TeSR1 medium is characterized by high concentration of FGF2 (100 ng/ml) to inhibit cell differentiation and promote self-renewal and pluripotency [39]. FGF2 is known to indirectly inhibit BMP signaling and, therefore, a lower concentration or absence of FGF2 would theoretically enhance the BMP-induced response. Furthermore, Transforming Growth Factor β1 (TGFβ1) (0.6 ng/ml) present in mTeSR1 activates SMAD2/3 pathway to help maintain hES cells in a proliferative state [40]. SMAD2/3 is known to form a complex with SMAD4, which is also a component of BMP-induced SMAD1/5/8 pathway, thus potentially interfering with BMP-induced differentiation [40]. In order to examine if BMP-induced differentiation of hES cells is affected by FGF2 and TGFβ1 present in the culture medium, we planned to test whether depletion of FGF2 and TGFβ1 would affect the percentage, speed or amount of differentiation to a particular fate in our culture conditions, using BMPs as inducers of differentiation.

Cells were split in mTeSR1 and incubated with a modified composition of mTeSR1 starting 24 h after splitting. The standard mTeSR1 culture medium, containing both FGF2 and TGFβ1 as described above, was modified to a composition lacking FGF2 or lacking both FGF2 and TGFβ1. Cells were maintained in these modified composition media for a total of 9 days, including a splitting process at day 4 of incubation. Treatments with BMPs in modified composition mTeSR1 started at day 5 of incubation in modified composition media and lasted for 4 days.

**Figure 7:**
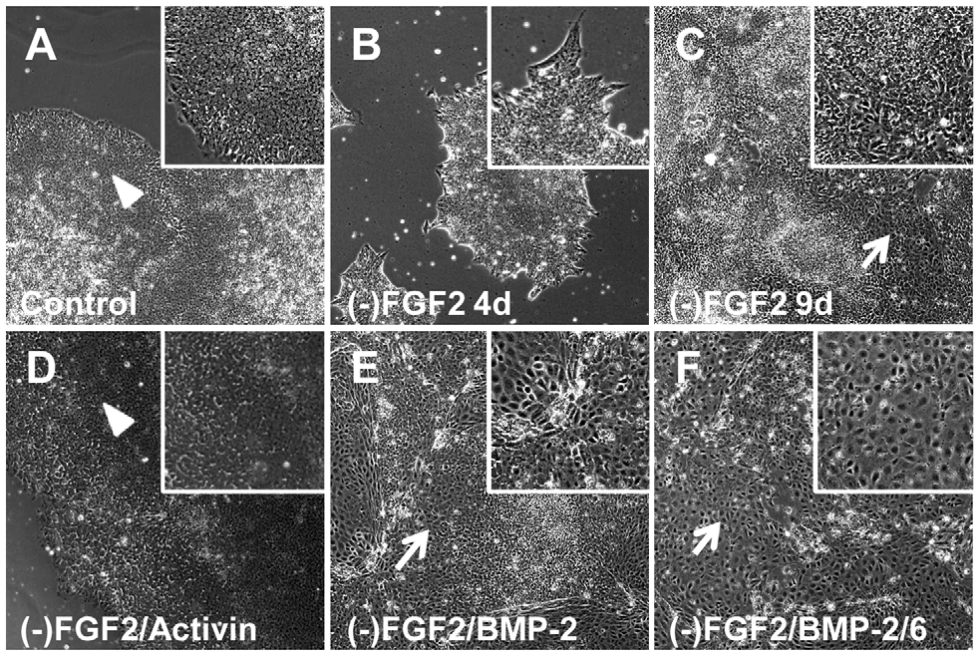
Morphological changes of hES cells after treatment with BMPs in FGF2-free mTeSR1. H9 cells were split in regular mTeSR1 and changed to FGF2-free mTeSR1 24 h after splitting. After 4 days of growing in modified composition media, colonies were split in FGF2-free mTeSR1 and incubated for other 5 days (total of 9 days). Treatments with BMP-2 or BMP-2/6 at 100 ng/ml started 24 h after splitting and lasted for 4 days. Pictures were taken using an inverted microscope and 10X objective. A, control cells after 9 days of culture in mTeSR1. B, Incubation in FGF2-free medium ((-)FGF2) for 4 days. C, Incubation in (-)FGF2 for 9 days. D, Treatment with 100 ng/ml Activin A in (-)FGF2 for 9 days. E, Treatment with 100 ng/ml BMP-2 in (-)FGF2 for 4 days. F, Treatment with 100 ng/ml BMP-2/6 in (-)FGF2 for 4 days. Arrowheads indicate morphologically pluripotent cells; arrows point out morphologically differentiated areas. Insets belong to 3× magnification.

After 4 days of incubation in FGF2-free medium, the effect of FGF2 depletion was observed as a change in the morphology of the colonies (star-shaped instead of rounded) and lower cell density (Figure 7B). When the colonies were split in FGF2-free medium, an elevated percentage of differentiated cells were observed in the following days (Figure 7C), to the point that cells cultured in FGF2-free mTeSR1 were unable to maintain pluripotency for more than two passages. Treatment with BMP-2 or BMP-2/6 at 100 ng/ml in FGF2-free medium for 4 days exacerbated differentiation triggered by the medium itself (Figure 7E, 7F), while treatment with Activin A at 100 ng/ml completely blocked differentiation (Figure 7D).

**Figure 8:**
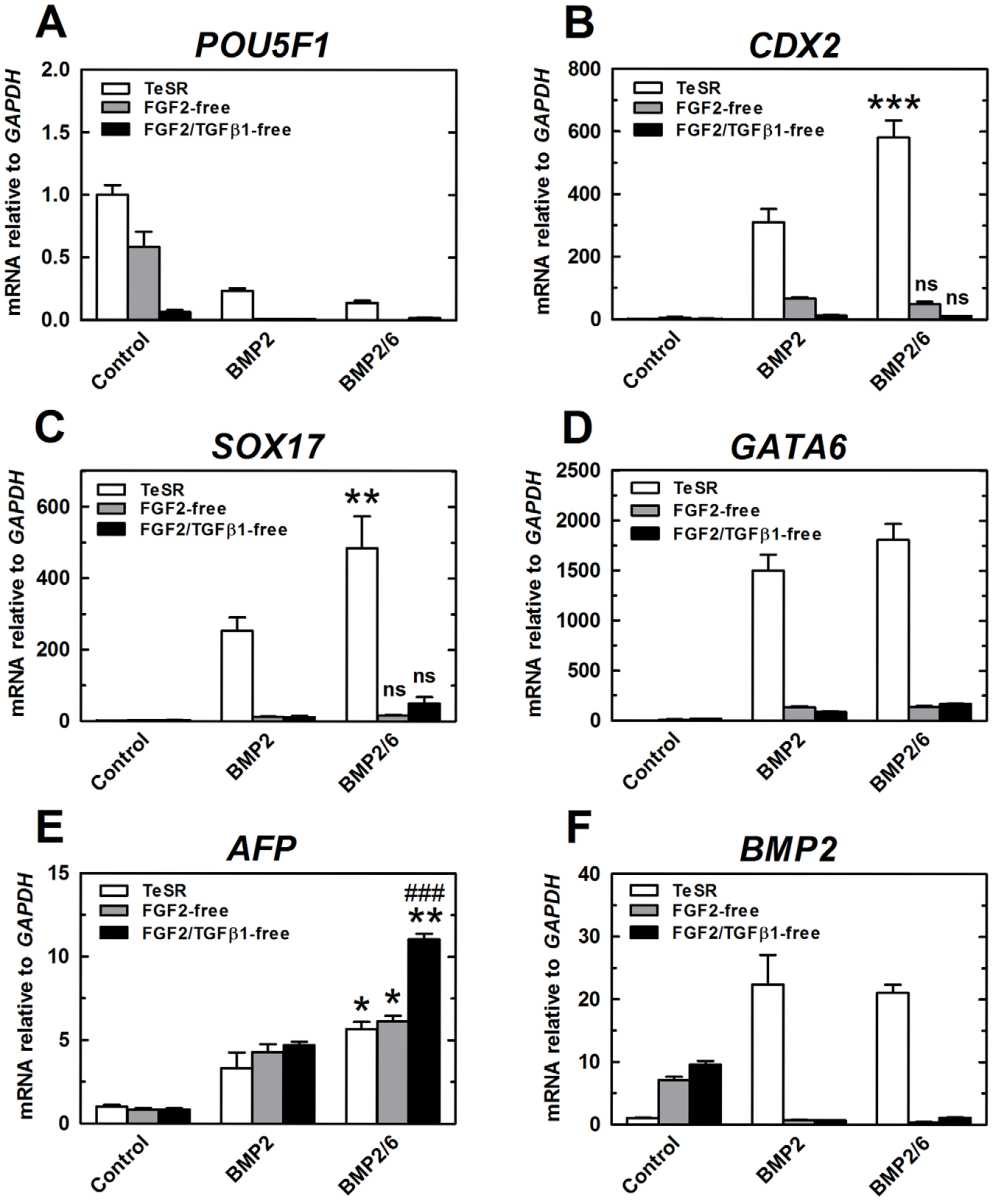
qPCR expression analysis of differentiation markers after treatment with BMPs in modified composition mTeSR1. H9 cells were split in regular mTeSR1 and changed to modified composition media 24 h after splitting. After 4 days of growing in modified composition media, colonies were split in the same modified composition medium and incubated for other 5 days (total of 9 days). Treatments with BMP-2 or BMP-2/6 at 100 ng/ml started 24 h after splitting in modified composition media and lasted for 4 days. qPCR was used to analyze expression of stem cell and differentiation markers. qPCR values correspond to relative expression compared to *GAPDH* mRNA. As control, cells growing in regular or modified composition mTeSR1 were used. Treatments were repeated at least in three different experiments, and results are expressed as average ± SD. A, *POU5F1*. B, *CDX2*. C, *SOX17*. D, *GATA6*. E, *AFP*. F, *BMP2*. (*) P<0.05 BMP-2 vs. BMP-2/6; (**) P<0.01 BMP-2 vs. BMP-2/6; (***) P<0.001 BMP-2 vs. BMP-2/6; ns, no significative difference BMP-2 vs. BMP-2/6; (###) P<0.001 control vs. BMP-2/6.

*POU5F1* expression was reduced by 50% after 9 days of incubation in FGF2-free medium and by 90% if the incubation was performed in FGF2/TGFβ1-free medium (Figure 8A). Incubation of H9 cells in FGF2-free or FGF2/TGFβ1-free media did not induce the expression of high levels of any of the differentiation markers analyzed, although differentiation was evident at the end of the treatment (Figure 7, Figure 8). We only detected an increased expression of *BMP2* in modified composition media (Figure 8F). These observations suggest that FGF2 and TGFβ1 are acting as anti-apoptotic, proliferative and pluripotency maintenance factors, but not inhibiting endogenous BMP-induced differentiation into ectodermal and endodermal lineage.

Next, we tested the differentiation-inducing activity of BMPs in these culture conditions. H9 cells cultured for 5 days in FGF2-free or FGF2/TGFβ1-free media were exposed to BMP-2 or BMP-2/6 at 100 ng/ml for 4 subsequent days in the modified composition media. When cells were treated with BMP-2 or BMP-2/6 after 5 days of FGF2 or FGF2/TGFβ1 depletion, the effect of those BMPs was characterized by an increased inhibition of *POU5F1* expression (Figure 8A); however, the expression of trophectodermal and endodermal markers was drastically abolished (Figure 8B–D). With the only exception of *AFP* marker (Figure 8E), BMP-2/6 and BMP-2 exerted the same effect on hES cells cultured in FGF2-free medium.

**Figure 9:**
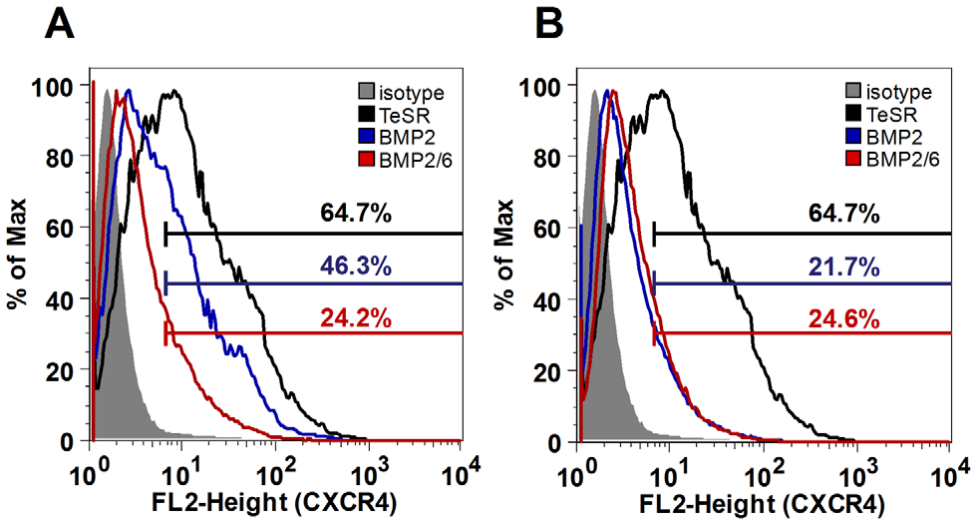
Expression analysis of endodermal marker CXCR4 in hES cells treated with BMPs in FGF2/TGFβ1-free mTeSR1. H9 cells were split in regular mTeSR1 and changed to FGF2/TGFβ1-free mTeSR1 24 h after splitting. After 4 days of growing in modified composition media, colonies were split in FGF2/TGFβ1-free mTeSR1 and incubated for other 5 days (total of 9 days). Treatments with BMP-2 or BMP-2/6 at 100 ng/ml started 24 h after splitting and lasted for 4 days. Flow cytometry was used to analyze expression of the endodermal marker CXCR4. As control, cells growing in FGF2/TGFβ1-free mTeSR1 (TeSR) were used. As negative control, PE-conjugated mouse IgG2a isotype control (isotype) was used. A, Flow cytometry histograms of CXCR4-positive cells after incubation with BMPs 10 ng/ml. B, Flow cytometry histograms of CXCR4-positive cells after incubation with BMPs 100 ng/ml. Bars indicate the percentage of CXCR4-positive cells in each condition.

These results were confirmed by flow cytometry for the endodermal marker CXCR4 (Figure 9). When incubated in a FGF2/TGFβ1-free medium, BMP-2 or BMP-2/6 diminished the percentage of CXCR4-positive cells, both at 10 ng/ml (Figure 9A) and at 100 ng/ml (Figure 9B) concentration. Therefore depletion of FGF2 in mTeSR1 did not enhance differentiation-inducing properties of BMP-2 or BMP-2/6, nor accentuated differences between them. These results suggest that FGF2, rather than block, synergistically acts with BMP signaling on the early stages of differentiation of hES cells to trophoblast and endoderm derivatives.

In light of the experimental results presented here, we can conclude that BMP-2/6 is a better inductor of differentiation of hES cells than BMP-2, and it's a good candidate for differentiation-guidance molecule. Furthermore, we demonstrate that FGF2 can directly regulate BMP-induced differentiation *in vitro*.

## Discussion

hES cells constitute a valuable model system in developmental studies and in the search for new regenerative therapies. Various stem cell types utilize BMP signals in a multitude of ways in order to define their fates [41]. BMPs are known to be involved in several types of differentiation processes and the use of BMPs in differentiation of pluripotent cells is a powerful tool in biological and medical research [32], [42]. The development of new molecules with increased activity is, therefore, a priority in medical and pharmaceutical research. The use of these BMPs with increased activity would permit the development of more efficient protocols and increase the productivity of the existent ones.

In this line of investigation, increased activity of BMP heterodimers has been previously reported; co-expression of BMP-2 with BMP-6 or BMP-7 yields heterodimeric BMPs with a specific activity about 20-fold higher than BMP homodimers in *in vitro* alkaline phosphatase induction assay, and 5- to 10-fold more potent than BMP-2 in inducing cartilage and bone in an *in vivo* assay [15], [19]. Other heterodimers, as BMP-4/7 or BMP-7/GDF-7, also showed enhanced and/or novel properties in the context of embryo development [16]–[18]. Therefore, BMP heterodimers could be used as a natural choice for substituting BMP homodimers in *in vitro* or *in vivo* assays. The production of BMP heterodimers in an *Escherichia coli* cell expression system and subsequent chemical refolding *in vitro* allowed us to work with high concentrations of purified protein while avoiding the low yield problems associated with a mammalian cell expression system [20].

The BMP-2/6 heterodimer consists of one BMP-2 and one BMP-6 subunit. Our experiments confirm the increased activity of BMP-2/6 heterodimer relative to the homodimeric counterparts BMP-2 or BMP-6, which support the hypothesis that such heterodimeric forms are likely to have natural biological functions. Previously BMP-2/7 heterodimer failed to exert an increased effect on hES cells growing on MEFs when compared to BMP-2 [9], indicating that culture conditions and/or conditioned media could play an important role in determining the fate of BMP-differentiated cells.

BMP-2 and BMP-6 are both inducers of osteogenesis in human mesenchymal stem cells [7], [8] and endogenous BMP-2 and BMP-6 cooperatively play pivotal roles in bone formation under both physiological and pathological conditions [43]. Nevertheless, type I and type II receptor utilization differs significantly between BMP-2/BMP-4 and BMP-6/BMP-7. BMP-4 is known to bind to BMPR1A and/or BMPR1B, whereas BMP-6 and BMP-7 preferentially bind to ACVR1 [44]. Also a greater reliance of BMPR2 is observed for BMP-2 (or BMP-4) relative to BMP-6 (or BMP-7), whereas ACVR2A is more critical to BMP-6 for signaling [45]. Unlike the homodimers, we recently reported that BMP-2/6 heterodimer exhibits a relatively high affinity for each receptor type extracellular domain (ECD) as measured by surface plasmon resonance [20]. Correlated to this, in several *in vitro* experiments we have observed stronger activity of BMP-2/6 than that of BMP-2 or BMP-6 [20]. Thus, the more potent inducer activity of BMP-2/6 on hES cells is also likely derived from higher affinity to both type I and type II receptors. In the present study we have observed an increased expression of *BMPR2* that could be responsible for the stronger effect of BMP-2 when compared to BMP-6.

For differentiation analysis we used a combination of markers of the three embryonic layers: ectoderm, mesoderm and endoderm. None of the BMPs we examined exclusively direct differentiation to only one cell type, but rather alter the relative proportions of a specific cell type. By quantification of the expression of differentiation markers, we identified BMP-2/6 as a better inducer of trophoblast and endoderm differentiation of hES cells than BMP-2 or BMP-6. BMP-2/6 is more effective than either BMP-2 or BMP-6 in inducing the expression of trophectodermal (*CDX2*), mesendodermal (*T*), and endodermal (*SOX17*, *GATA6*, *AFP*, *CXCR4*) markers, including the expression of BMPR2 receptor. These results support an increased bioactivity of BMP-2/6 in the described stem cell culture system, and it is being confirmed using other hES cell lines.

The increase in *CDX2* expression indicates trophectodermal differentiation and suggests that in feeder-free conditions BMP-2 induces trophoblast, as observed previously with BMP-4 [46]. Markers of endoderm (both extraembryonic and definitive) were also strongly induced. The definitive endoderm forms during gastrulation and replaces the extraembryonic visceral endoderm. Definitive endoderm is the precursor of several organs (pancreas, liver) and *in vitro* differentiation to those cell types is of great use in medical and biological studies. To generate definitive endoderm, 100 ng/ml of Activin A has been traditionally used in unconditioned medium [33], revealing a central role of TGFβ family pathways on this type of differentiation. Feeder layer-free conditions are also better systems for differentiation of hES cells into definitive endoderm [30]. Our results demonstrate that the heterodimer BMP-2/6 is better inductor of endodermal markers than the BMP-2 homodimer, including definitive endoderm. It suggests that BMP-2/6 could replace BMP-2 as the inductor in protocols of BMP-2-guided differentiation *in vitro*.

BMP receptors are present on hES cells and BMP signaling can induce expression of BMP ligands, forming a positive feedback loop in cells from various species, including human [47]. Specifically, BMP-2 can induce its own expression in human embryonic carcinoma cells [9]. Treatment of human pluripotent cells with BMP-2 leads to the accumulation of transcripts for this factor consistent with a positive feedback model [9], but an increased level of *BMP2* gene transcription in hES cells had not been reported. In our experiments we observed an increased expression of both *BMP2* and the type II receptor *BMPR2* in cells treated with BMP-2 and BMP-2/6. Up-regulation of the expression of both ligand and receptor is a strong evidence of a positive feedback of BMP-2 signaling and differential induction of *BMPR2* could be also a factor in the increased biological activity of BMP-2/6 in hES cells. These results should be confirmed in the future by analyzing levels of both proteins in hES cells after BMP treatment.

BMPs play an important role during all stages of embryonic development, and although only two major signaling pathways have been characterized (p38 and Smad pathways), the BMP signaling is complex and includes cross-talk with other major signaling pathways and negative feedback mechanisms [34], [48], [49]. It has been reported that the initiation of Smad-dependent and Smad-independent signaling by BMP-2 depends on BMP-receptor complexes [37], [50], [51]. BMP-2/6 possesses a higher affinity to both receptors type I and II than its homodimeric counterparts [20], and this new biological property could be responsible for the increased activation of Smad-dependent and Smad-independent signaling observed. We detected increased levels of SMAD1/5 phosphorylation induced by BMP-2/6, which confirms the recent reports using diverse experimental models, as luciferase reporter assay, osteogenic differentiation-inducing activity and chondrogenic activity [20]. Furthermore, increases in SMAD1 phosphorylation have been previously reported in the early events of BMP-induced hES cell differentiation [9], [40]. Further study of BMP-2/6-activated signaling pathways would be necessary to fully understand its increased potency in hES cell differentiation.

Fibroblasts secrete multiple growth factors, including FGFs, TGFβs, Activins, Wnts and antagonists of BMP signaling [6]. Of those, FGF2 has the greatest effect in promoting hES cell self-renewal, interrupting BMP signaling either by preventing the nuclear translocation of phosphorylated SMAD1 [52] or by repressing SMAD1 activity in the nucleus [53]. Suppressed BMP signaling remains a consistent hallmark of current methods of hES cell culture. When we cultured H9 cells in a modified TeSR1 medium without FGF2, cells differentiated quickly after splitting and this differentiation was not blocked by noggin (data not shown), but blocked by 100 ng/ml Activin A, suggesting a compensatory effect due to the activation of the SMAD2/3 pathway [54]. Depletion of FGF2 has a more drastic effect than TGFβ1, as FGF2 is also involved in cell proliferation [46] and trace levels of TGFβ1 are present in Growth Factor-Reduced Matrigel coating. Our results confirm that both FGF2 and TGFβ1 in mTeSR1 are necessary for long term maintenance of H9 cells in mTeSR1. Further analysis of this involvement should be performed to improve and manipulate BMP-directed differentiation of hES cells *in vitro*.

FGF2 depletion also affected BMP-2 and BMP-2/6-induced differentiation by diminishing levels of expression of all the differentiation markers analyzed, both trophectodermal and endodermal. Presence of FGF2 was necessary for driving BMP-2 or BMP-2/6-induced differentiation to endoderm in mTeSR1. When FGF2 was absent in mTeSR1, however, morphological differentiation appeared but the pattern of expression of differentiation markers after incubation with BMP-2 or BMP-2/6 completely changed. We hypothesize that FGF2 inhibition of neural differentiation could be a requirement for efficient BMP-induced differentiation to endoderm. Further investigation in this sense is required to clarify the involvement of FGF2 in BMP-induced differentiation in mTeSR1.

We analyzed the biological activity of BMP-2/6 for inducing differentiation of hES cells by measuring the expression of differentiation markers using qPCR and flow cytometry. Both BMP-2 and BMP-6 induce differentiation of hES cells, but the heterodimer BMP-2/6 is a more efficient inductor of expression of differentiation markers and percentage of CXCR4-positive definitive endoderm cells. It suggests that BMP-2/6 is a better candidate than BMP-2 as inductor in protocols of BMP-2-guided differentiation *in vitro*, as well as possible applications of BMP-2/6 to treat bone injury substituting BMP-2 as active molecule.

## Materials and Methods

### Human embryonic stem cell culture

hES cell line H9 (WiCell, WI) was cultured in mTeSR1 (StemCell Technologies) on Growth Factor-Reduced Matrigel (BD Biosciences) following the established protocol [55]. When colonies reached 80–90% confluence, they were detached of the plates with dispase 2 mg/ml in DMEM/F12 (1∶1) (Invitrogen), washed with DMEM/F12 and then scrapped in mTeSR1 and split 1∶4 to 1∶6 in Matrigel coated wells. Medium was changed daily for fresh mTeSR1. For experiments, passages 40–60 were used.

### Protein expression and purification

The mature domains of human BMP-2 (residues 1–110), human BMP-6 (residues 1–132) and noggin were expressed in *Escherichia coli* as inclusion bodies. The expressed inclusion bodies were isolated, purified, and refolded using a modified protocol [12], [20], [56], [57]. The refolded BMP-2 and BMP-6 homodimers and BMP2/BMP6 heterodimer were purified using a HiTrap heparin column (GE Healthcare, Uppsala, Sweeden) and reversed phase chromatography (GraceVydac, Deerfield, IL). The ligands were lyophilized and resuspended in 10 mM HCl and stored at −80°C (supplied by joint Protein Central, http://www.jointproteincentral.com). After thawing, compounds can be stored at 4°C for one week to one month. The biological activity of media supplemented with BMPs was not affected after storage at 4°C for one month.

### Treatment with agonists and modified media

For treatments in regular medium, mTeSR1 supplemented with the desired concentration of agonists (1–100 ng/ml) was prepared, filtered and daily added to the culture. For modified composition media, mTeSR1 was prepared as previously reported [55] by mixing all its components but the ones to be depleted. This medium was supplemented with human recombinant FGF2 (Invitrogen) and/or TGFβ1 (R&D Systems) if necessary for control condition. Pictures were taken using an Olympus IX51 inverted microscope and QuantiFire XI Cooled Digital CCD Camera and processed using PictureFrame software.

### RNA extraction and reverse transcription reaction

Total RNA was extracted following the guanidinium thiocyanate-phenol-chloroform protocol [21] using TRIzol reactive (Invitrogen). 5 µg of total RNA were used for reverse transcription reaction, and mRNA was converted to complementary DNA (cDNA) using oligo dT primers and Superscript II reverse transcriptase (Invitrogen). For qPCR analysis 2 µl of total cDNA were diluted in 90 µl of H_2_O, and 4 µl of this dilution were used in each reaction.

### Quantitative PCR (qPCR) analysis

Primers for qPCR were designed using Primer3 software [58] to yield a 75–150 bp product, 20 bp long and Tm 60°C. Sequences of primers, location, Tm and GC% are available in Table 1.

**Table 1:** qPCR primers of human genes.

Gene	OLIGO	START	Tm	GC%	SEQUENCE
*ACVR1*	LEFT PRIMER	555	60.00	55	GCGGTAATGAGGACCACTGT
	RIGHT PRIMER	660	60.07	50	CCCTGCTCATAAACCTGGAA
*ACVR2A*	LEFT PRIMER	519	59.97	50	ACACAGCCCACTTCAAATCC
	RIGHT PRIMER	662	59.96	55	AGGAGGGTAGGCCATCTTGT
*ACVR2B*	LEFT PRIMER	9596	60.00	55	CATGGGACACAAGGTCAGTG
	RIGHT PRIMER	9738	60.00	55	GGCAGTGCTCTGAGAAAACC
*AFP*	LEFT PRIMER	1713	60.03	45	CTTGTGAAGCAAAAGCCACA
	RIGHT PRIMER	1834	60.13	55	CCCTCTTCAGCAAAGCAGAC
*BMP2*	LEFT PRIMER	1631	59.89	45	TCAAGCCAAACACAAACAGC
	RIGHT PRIMER	1733	59.93	50	AGCCACAATCCAGTCATTCC
*BMP6*	LEFT PRIMER	1141	59.96	50	ACAGCATAACATGGGGCTTC
	RIGHT PRIMER	1252	60.02	55	GAAGGGCTGCTTGTCGTAAG
*BMPR1A*	LEFT PRIMER	898	59.87	50	AGCTACGCCGGACAATAGAA
	RIGHT PRIMER	978	59.99	55	CTATGACAACAGGGGGCAGT
*BMPR1B*	LEFT PRIMER	239	59.98	55	GCCTGCCATAAGTGAGAAGC
	RIGHT PRIMER	320	59.97	45	CTTTCTTGGTGCCCACATTT
*BMPR2*	LEFT PRIMER	1421	60.02	55	GGTAAGCTCTTGCCGTCTTG
	RIGHT PRIMER	1526	60.04	45	ATCTCGATGGGAAATTGCAG
*CDX2*	LEFT PRIMER	1738	60.05	55	AGGGGGTGGTTATTGGACTC
	RIGHT PRIMER	1829	60.1	55	CATTCAGCCCAGAGAAGCTC
*CGB5*	LEFT PRIMER	510	60.01	55	GTCAACACCACCATCTGTGC
	RIGHT PRIMER	603	59.6	55	GGTAGTTGCACACCACCTGA
*CXCR4*	LEFT PRIMER	557	60.00	55	GGTGGTCTATGTTGGCGTCT
	RIGHT PRIMER	632	60.02	50	CTCACTGACGTTGGCAAAGA
*GAPDH*	LEFT PRIMER	48	60.02	50	ACAGTCAGCCGCATCTTCTT
	RIGHT PRIMER	141	59.97	50	ACGACCAAATCCGTTGACTC
*GATA4*	LEFT PRIMER	2446	60.02	45	AAATGCAGCTGGCAACTTCT
	RIGHT PRIMER	2546	60.03	45	AGCGGGAAGAGGGATTTTTA
*GATA6*	LEFT PRIMER	2099	60.02	50	TCCACTCGTGTCTGCTTTTG
	RIGHT PRIMER	2238	60.01	55	CCCTTCCCTTCCATCTTCTC
*NANOG*	LEFT PRIMER	377	60.00	50	TTCCTTCCTCCATGGATCTG
	RIGHT PRIMER	451	60.01	45	AAGTGGGTTGTTTGCCTTTG
*NESTIN*	LEFT PRIMER	2077	60.04	45	TCCAGGAACGGAAAATCAAG
	RIGHT PRIMER	2196	60.04	60	GCCTCCTCATCCCCTACTTC
*POU5F1*	LEFT PRIMER	774	59.99	55	AGTGAGAGGCAACCTGGAGA
	RIGHT PRIMER	883	59.97	55	ACACTCGGACCACATCCTTC
*SOX17*	LEFT PRIMER	2170	59.17	45	CCAGAGGCTTTTTGGATGTT
	RIGHT PRIMER	2272	59.97	50	GCCACTTCCCAAGGTGTAAA
*T*	LEFT PRIMER	1902	59.96	45	AAGAAGGAAATGCAGCCTCA
	RIGHT PRIMER	2002	60.05	55	TACTGCAGGTGTGAGCAAGG

PCR reactions were prepared in microamp optical 96-well reaction plates (Applied Biosystems). 4 µl of a 1∶45 dilution of total cDNA were mixed with 5 µl SYBR green PCR master mix 2X (Roche) and 1 µM of each primer pair in a total volume of 10 µl. Reactions were run in an ABI Prism 7900 Sequence Detector (Applied Biosystems) and results analyzed with SDS2.3 software (Applied Biosystems) for Ct calculations. Calculations of ΔΔCt values were performed following specifications of the manufacturer.

### Flow cytometry of human embryonic stem cells

H9 cells were treated with BMP-2, BMP-6 or BMP-2/6 at 10 ng/ml or 100 ng/ml for 5 days and then digested into individual cells with TrypLE (Sigma) diluted 1∶4 in PBS (Invitrogen) for several minutes at 37°C. Cell suspension was incubated with fluorophore-tagged primary antibody or isotype control in phosphate-buffered saline (PBS) with 2% fetal bovine serum. For quantifying pluripotent embryonic stem cells, Alexa Fluor 488 mouse anti-human TRA-1-60 (BD Biosciences) was used. For labeling definitive endoderm cells, Phycoerythrin (PE)-conjugated mouse monoclonal anti-human CXCR4 (R&D Systems) or PE-conjugated mouse IgG2a isotype control (eBioscience) were used. Cells were sorted using a Becton-Dickinson FACScan analytical flow cytometer and data were analyzed with FlowJo software.

### Immunoblot analysis

H9 cells treated with BMP-2, BMP-6 or BMP-2/6 at 100 ng/ml for 5, 10, 30, 60 or 120 min were washed with PBS, and solubilized in RIPA buffer containing 50 mM Tris-HCl pH7.5, 150 mM NaCl, 1% NP-40, 0.1% SDS and 0.5% sodium deoxycholate. Lysates were subjected to SDS-gel electrophoresis, and proteins were electrotransferred to polyvinylidene difluoride membranes and immunoblotted with specific antibodies. All the antibodies used were from Cell Signaling Technologies (1∶1000), except anti-ERK (pan ERK) from BD Transduction Laboratories (1∶20000). Secondary HRP-conjugated antibodies were from Bio-Rad. Labeled proteins were visualized using an enhanced chemiluminescence detection system (Thermo Scientific).

### Statistical analysis

Statistical analysis was performed using GraphPad Prism 5 software. One-way ANOVA and Bonferroni post-test were used to compare multiple data sets, and t-Student test was used to compare two data sets when necessary.

## Supporting Information

Figure S1 qPCR expression analysis of BMP receptors and agonist after treatment with BMPs. H9 cells were treated with BMP-2, BMP-6 or BMP-2/6 at 100 ng/ml in mTeSR1 for 5 days. After 5 days of treatment, qPCR was used to analyze expression of BMP receptor and agonists. qPCR values correspond to relative expression compared to *GAPDH* mRNA. As control, cells growing in mTeSR1 were used. Treatments were repeated at least in three different experiments, and results are expressed as average ± SD. A, *BMPR1*. B, *BMPR1B*. C, *ACVR1*. D, *ACVR2A*. E, *ACVR2B*. F, *BMP6*.
(0.68 MB TIF)
