# Supplementary figures and images for: BMP-2/6 Heterodimer Is More Effective than BMP-2 or BMP-6 Homodimers as Inductor of Differentiation of Human Embryonic Stem Cells
Source: PLoS One. 2010 Jun 17;5(6):e11167. doi: 10.1371/journal.pone.0011167 (PMC2887366; doi:10.1371/journal.pone.0011167)

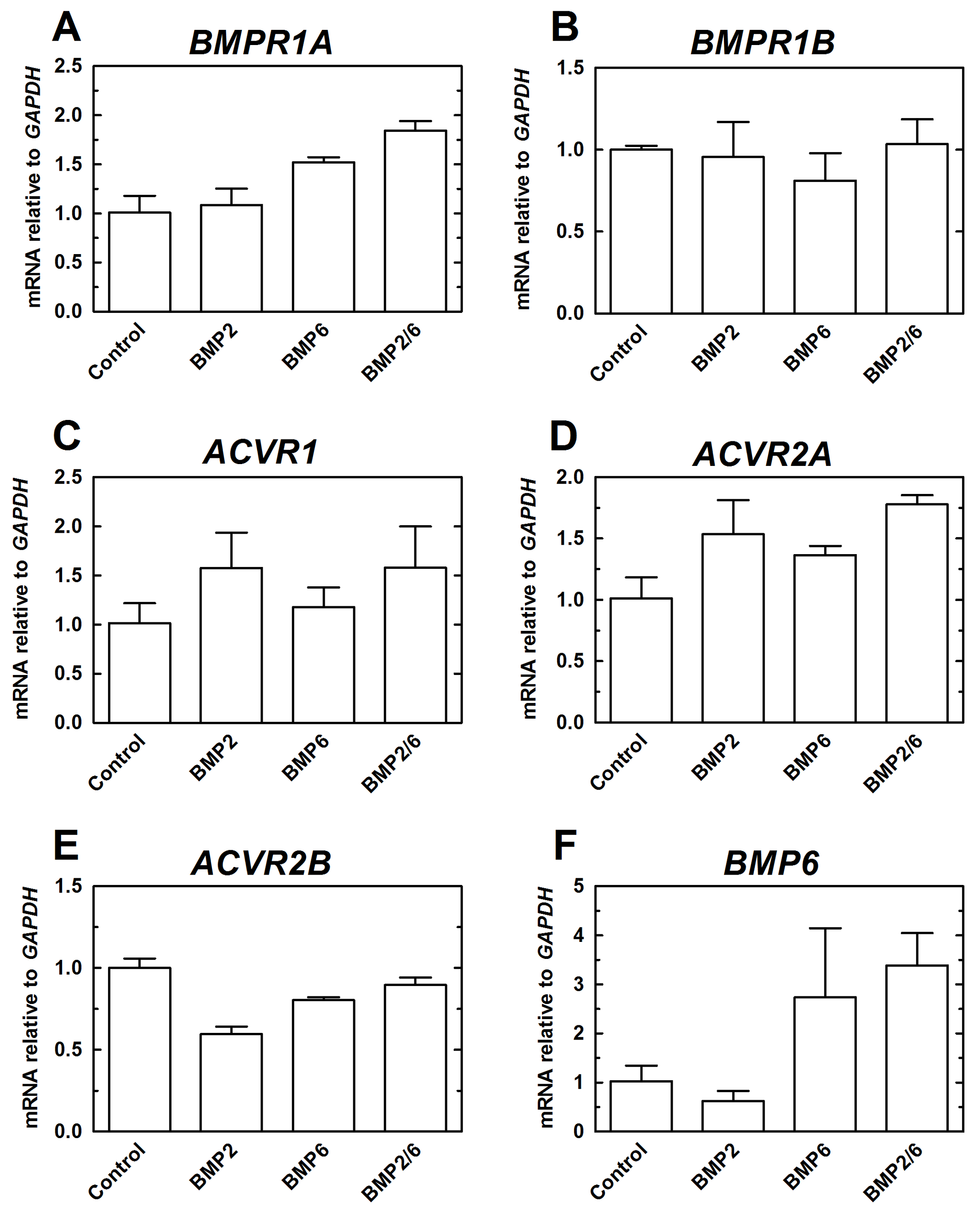

Supplement: Figure S1 — qPCR expression analysis of BMP receptors and agonist after treatment with BMPs. H9 cells were treated with BMP-2, BMP-6 or BMP-2/6 at 100 ng/ml in mTeSR1 for 5 days. After 5 days of treatment, qPCR was used to analyze expression of BMP receptor and agonists. qPCR values correspond to relative expression compared to GAPDH mRNA. As control, cells growing in mTeSR1 were used. Treatments were repeated at least in three different experiments, and results are expressed as average ± SD. A, BMPR1. B, BMPR1B. C, ACVR1. D, ACVR2A. E, ACVR2B. F, BMP6. (0.68 MB TIF) [file pone.0011167.s001.tif]
